# ACCEPTABLE CHARACTERISTICS OF INTERVENTIONS FOR MIDDLE-AGED HISPANIC MEN: RESULTS FROM KEY INFORMANT INTERVIEWS

**DOI:** 10.1093/geroni/igz038.521

**Published:** 2019-11-08

**Authors:** Cheryl DerAnanian, Sonia Vega-Lopez, Hector Valdez, Shandel Vega-Soto, Ferdinand Delgado, Steven Hooker

**Affiliations:** 1 Arizona State University, Phoenix, Arizona, United States; 2 Arizona State University, Phoenix, United States; 3 San Diego State University, San Diego, California, United States

## Abstract

Hispanics are the fastest growing segment of the aging population. Little is known about acceptable intervention approaches for Hispanic men. Purpose: To explore perceptions of acceptable intervention characteristics for middle-aged Hispanic men. Methods: Eighteen key informants (KIs) with expertise in delivering programs to the Hispanic community participated in semi-structured interviews. All interviews were audio-recorded and transcribed verbatim. Themes were identified using a grounded theory approach. Results: “Cultural sensitivity/competency” or “biculturalism” were fundamental characteristics of an acceptable intervention (n=17 KIs). KIs indicated program facilitators need to understand and relate to the culture, and to establish the trust of the community by demonstrating mutual understanding, respect and dignity. Facilitators must be bilingual and use the same level of language as the participants. KIs highlighted the importance of the literacy level of the materials and indicated intervention content should tap into the participants’ unique cultural experiences. Including the family in the intervention, especially the spouse or partner, was considered essential for engaging the men (n=18 KIs). The KIs consistently indicated an intervention should be in-person, in either a group (n=7 KIs), one-on-one (n=5 KIs) or hybrid (group and individual; n=6) format. Only text messaging (n=10 KIs), phone calls (n=5 KIs), and Fitbits (n= 7KIs) were perceived as acceptable forms of technology for an intervention. Finally, the intervention needs to be appealing (N=5KIs), interactive (n=8KIs), and safe, fun and supportive. Conclusion: The KIs identified several important culturally relevant considerations when developing interventions for Hispanic men. Funded by the National Institute on Aging (R21 AG050084-01A1).

